# Reports on the Hospitals of the United Kingdom

**Published:** 1911-11-18

**Authors:** Henry Burdett


### REPORTS ON THE HOSPITALS OF THE UNITED KINGDOM.

By SIR HENRY BURDETT, K.C.B., K.C.V.O.

### LVII.—Johnson Hospital, Spalding.

This Hospital was established 31 years ago. It was erected from special designs so recently as 1881, was handsomely equipped, and money was, and apparently is, still amply forthcoming. It is the most recent of modern hospitals and proves to demonstration how rapidly an endowed hospital in these days may become neglected and inefficient. Its condition in many respects at the present time should surely decide any millionaire who might inspect it that the day for endowing hospitals in this country is passed and gone if efficiency and up-to-dateness are regarded as primary considerations. It would seem as if the public took practically no interest whatever in this institute at the present time.

The trustees under the will of the founders, the Misses E. A. and M. A. Johnson, cannot surely have gone carefully over the whole of the hospital from top to bottom at least once a year, or the walls and several of the fittings could never have been allowed to fall, in the hospital sense, into their present disgracefully neglected condition. If the walls of the wards and corridors had been painted four coats, they might have stood the absence of any further expenditure for ten years at least. As the walls have simply been coloured for the most part, and then left without recolouring or any attempt to make them hygienically perfect and as smart in appearance as possible, they present a most melancholy spectacle, especially to those who know what ward and corridor walls ought to be in a hospital. Except for some recolouring which the porter has managed partially to do to some of the walls in the corridors, the whole appearance of the inside of this hospital is lamentable and grievous. This atmosphere of neglect is a constant factor, and even the seats of the w.c.s are all to pieces and not fit to use. It is not necessary for us to find the reason for this neglect, which is discreditable to every person responsible for the administration of its affairs. How discreditable, the audited accounts prove by showing that the trustees have upwards of £450 available. Had they been alive to the duties of a hospital trustee, responsible for the upkeep of a British hospital in the present day, this institution could never have been allowed to fall into its present disgraceful state of internal disrepair and shabbiness. The whole place bears a most depressing air of neglect, and it is time that steps are taken either to close the hospital or to reorganise it and make it worthy of the intentions and hopes of the estimable ladies who gave their money for its foundation and maintenance. The whole building requires overhauling from top to bottom, inside and out; the whole of the fittings should be put into a thorough state of repair and renewed where necessary; and the management should be so reconstituted as to provide guarantees that never again shall it be possible for this institution to experience similar structural neglect to that from which it is suffering so grievously to-day.

There were only a few patients in the hospital on the day of our visit, and we can well believe it to be possible that many patients and their friends do not care to seek admission to the wards in present circumstances. In saying this it must be understood that so far as we were able to judge the resident staff have done their best to keep the building clean and to do their work conscientiously, notwithstanding the depressing surroundings and conditions under which it has to be done owing to the supineness and indifference or neglect, or all three, of the trustees who must be credited with the responsibility. Unfortunately it has become probably the most neglected and unsatisfactory hospital building at present used anywhere in this country for the treatment of the sick and suffering. No staff, however efficient, can do their work properly unless the means are forthcoming to provide each year for the necessary repairs and maintenance. They should also be encouraged by the sympathy of the residents surrounding the hospital who recognise the duty of personal service in the days of health in the cause of the sick. Wake up, Spalding! wake up, trustees! and either close this neglected building or hasten to make it what it ought to be—a bright, up-to-date, modern hospital, and so a health asset for the whole population of Spalding, which it was founded to serve. We were struck with the gruff, not to say rude, reception we received from the porter on arrival, and we formed the conclusion that there were too many dogs about for a hospital, which, for various reasons, is better without them as residents.

